# Antiviral Activity of Oligonucleotides Targeting the SARS-CoV-2 Genomic RNA Stem-Loop Sequences within the 3′-End of the ORF1b

**DOI:** 10.3390/pathogens11111286

**Published:** 2022-11-01

**Authors:** Maria Alfreda Stincarelli, Arianna Rocca, Alberto Antonelli, Gian Maria Rossolini, Simone Giannecchini

**Affiliations:** 1 Department of Experimental and Clinical Medicine, University of Florence, I-50134 Florence, Italy; 2 Microbiology and Virology Unit, Florence Careggi University Hospital, I-50134 Florence, Italy

**Keywords:** nucleic-acid base therapy, SARS-CoV-2, Oligonucleotides, ORF1b RNA stem-loop packaging sequences, COVID-19

## Abstract

Increased evidence shows vaccines against severe acute respiratory syndrome coronavirus 2 (SARS-CoV-2) exhibited no long-term efficacy and limited worldwide availability, while existing antivirals and treatment options have only limited efficacy. In this study, the main objective was the development of antiviral strategies using nucleic acid-based molecules. To this purpose, partially overlapped 6-19-mer phosphorothioate deoxyoligonucleotides (S-ONs) designed on the SARS-CoV-2 genomic RNA stem-loop packaging sequences within the 3′ end of the ORF1b were synthetized using the direct and complementary sequence. Among the S-ONs tested, several oligonucleotides exhibited a fifty percent inhibitory concentration antiviral activity ranging from 0.27 to 34 μM, in the absence of cytotoxicity. The S-ON with a scrambled sequence used in the same conditions was not active. Moreover, selected 10-mer S-ONs were tested using different infectious doses and against different SARS-CoV-2 variants, showing comparable antiviral activity that was abrogated when the central sequence was mutated. Experiments to evaluate the intracellular functional target localization of the S-ON inhibitory activity were also performed. Collectively the data indicate that the SARS-CoV-2 packaging region in the 3′ end of the ORF1b may be a promising target candidate for further investigation to develop innovative nucleic-acid-based antiviral therapy.

## 1. Introduction

The origin of the COVID-19 pandemic was due to the new severe acute respiratory syndrome coronavirus 2 (SARS-CoV-2), related to betacoronaviruses [1,2,3]. Today it is increasingly evident that, although vaccines against SARS-CoV-2 are the main solution to counter the pandemic, their long-term efficacy and their worldwide availability is limited [4]. Moreover, reported emerging SARS-CoV-2 variants and new potential animal coronavirus transmission to the human population pose a concern.

Existing antivirals and treatment options against COVID-19, mainly made up of viral proteins, have shown only limited efficacy to date, raising the urgency the development of new strategies [5]. Although their role in viral life cycles is not completely clear, structural features of viral RNA sequences could be used in new antiviral target development [6,7,8,9]. It is of note that nucleic-acid based RNA vaccines expressing a spike protein containing modified nucleosides to suppress RNA-mediated immune activations and significantly enhance translation rates have demonstrated several advantages over traditional vaccines to combat outbreaks of SARS-CoV-2 due to their rapid development and high target specificity [10]. Some evidence has shown that the genomes of RNA viruses contain cis-acting RNA elements located at the 5′ and 3′ untranslated regions and in the coding region, forming stem-loop structures implicated in the RNA and viral or host proteins RNA–RNA or RNA–protein interactions to fulfil viral genome replication, translation, and assembly [11]. The genomic packaging sequences of the influenza virus, extensively characterized for their implications for viral assembly and propagation [12,13,14,15], have been used to develop new nucleic-acid-based antiviral strategies [16,17,18,19,20]. Although not as well studied as influenza virus, the SARS-CoV-2 RNA genomes possess similar features that mediate viral assembly and that are potentially targetable by nucleic-acid molecules [10,21,22,23,24,25,26]. The high conservation of the sequence of packaging in the SARS-CoV-2 genome suggests a potential for the maintenance of RNA structures possessing biological functionalities [23]. In this context, the antiviral activity of nucleic-acid-based molecules targeting the 5′ constant region of the SARS-CoV-2 genome has been proven [10,27,28,29]. An intriguing issue is that, similar to influenza virus and other RNA viruses, coronavirus defective interfering genomes that emerge during viral replication and counteract viral propagation present the functional packaging elements of the coronavirus genomes that are critical to the process of mature virion formation [30,31,32,33]. Thus, this has led to the suggestion that the SARS-CoV-2 RNA regions containing packaging signals might be suitable targets for antiviral action.

Here, it was considered of interest for the antiviral development strategies based on the use of nucleic acids to investigate the potential of oligonucleotide inhibitors targeting the packaging genomic regions of SARS-CoV-2. In this work, phosphorothioate deoxyoligonucleotides (S-ON) designed on a direct or complementary sequence of the SARS-CoV-2 genomic RNA stem-loop (SL)1 and SL2 packaging signals within the 3′ end of the ORF1b were synthetized and assayed against SARS-CoV-2 virus variants using different viral infectious doses. Moreover, experiments to evaluate the intracellular localization of the S-ON and a potential target of the inhibitory activity were carried out.

## 2. Materials and Methods

### 2.1. Cells and Viruses

The cell lines used were Vero E6 (BS-CCLO87, ATCC, Rockville, MD, USA) cultivated using DMEM culture medium supplemented with 10% fetal bovine serum (FBS). In some experiments, human breast adenocarcinoma cells (MCF-7, HTB-22, ATCC) transiently expressing human ACE2 receptor (pLENTI_hACE2_PURO expressing plasmid) (MCF7-hACE2) were used, maintained in modified RPMI supplemented with 10% FBS, and Madin–Darby canine kidney (MDCK; BS-CCL34, ATCC, Rockville, MD, USA), maintained in modified Eagle’s medium (MEM) supplemented with 10% FBS. The viruses used were SARS-CoV-2 clinical isolates (SCV2/Fi/3/22 Wuhan-like, SCV2/Fi/1/21 Alpha-like and SCV2/Fi/2/21 Delta-like variant) grown on Vero E6 cells and titrated by the plaque method. The influenza virus used was the A/Firenze/02/2019 H1N1pmd strain grown on MDCK cells and titrated by the plaque method. The viral stocks, consisting of cell-free supernatants of acutely infected cells, were aliquoted and stored at −80 °C until used.

### 2.2. Phosphorothioate Deoxyoligonucleotide Synthesis

**Figure 1:**
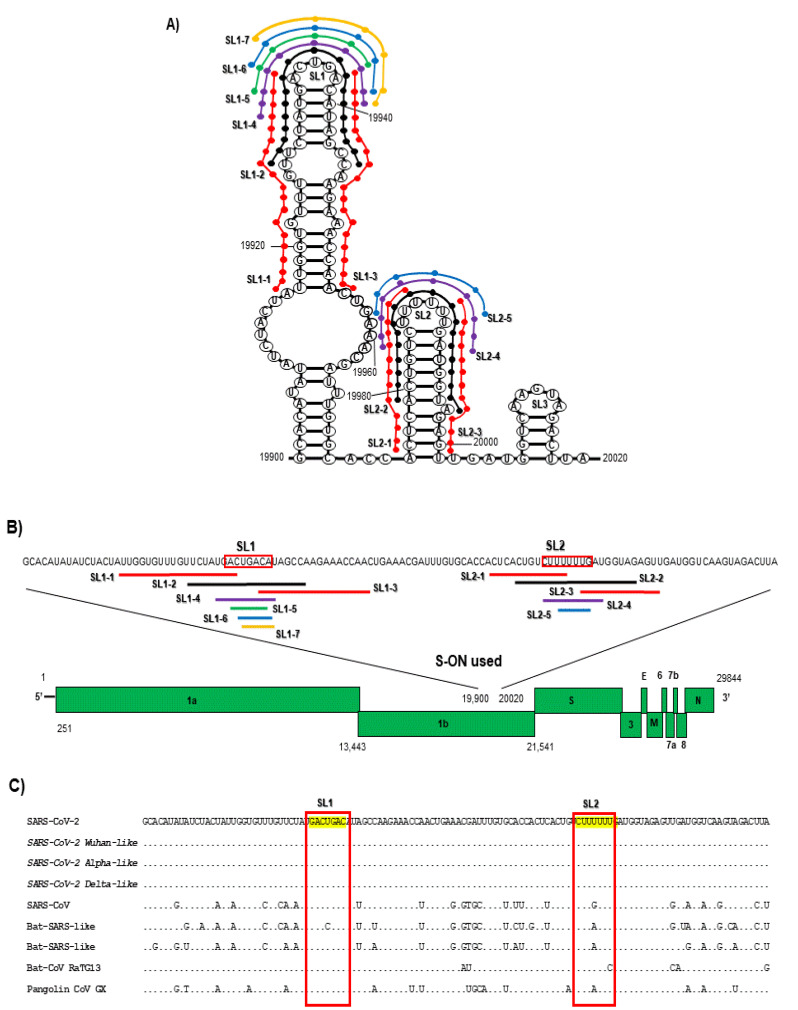
Schematic representation of the predicted RNA secondary structures (**A**) of the 3′ end of the ORF1b region of the genome (**B**) of SARS-CoV-2 and the localization of S-ONs (A, B) used in the study. The RNA secondary structure was obtained using Mfold structure-prediction system [34]. (**C**) Variability of the viral RNA 3′ end of the ORF1b region from 19,900 to 20,019 nucleotide for SARS-CoV-2, including SARS-CoV-2 type-like variants used in this study, SARS-CoV, bat SARS-like CoV, and pangolin CoV are also shown.

The phosphorothioate deoxyoligonucleotides (S-ON) were designed by selecting the SARS-CoV-2 genomic sequence of RNA stem-loop packaging sequence within the 3′ end of the ORF1b (Figure 1) and synthetized and purified according to traditional methods, using modifications to make them stable (Roche, Milan, Italy).

### 2.3. SARS-CoV-2 S-ON Inhibitory Assay

Unless otherwise stated, for the inhibitory activity against SARS-CoV-2 variants the selected S-ON were tested on 24-well plates containing Vero E6 cells using multiplicities of infection (MOI) of 0.01. After 1 h of incubation at 37 °C in humidified air with 5% CO_2_, the viral inocula were removed, and the cells were washed twice with PBS and treated with the S-ONs under study. To facilitate S-ON entry into the cells, Lipofectamine 2000 reagent (Invitrogen, Carlsbad, CA, USA) was used as a lipid-based carrier. Lipofectamine was diluted in DMEM (5%) and kept at room temperature for 5 min. The S-ONs, properly dissolved in DMEM without serum (the final concentration added to the cells ranged from 0.1 to 100 μM), were combined with lipofectamine, mixed gently, and kept at room temperature for 20 min. Transfection was carried out by adding 200 μL of the mixtures (S-ONs and lipofectamine) to each well containing cells and incubating at 37 °C in a CO_2_ incubator for 4 h. Virus treated with lipofectamine alone was used as control. The cells were then washed and replaced with fresh DMEM supplemented with L-1-tosylamido-2-phenylethyl-chloromethyl-ketone-treated trypsin (2 μg/mL; Sigma, St. Louis, MO, USA). The inhibitory activity was analyzed by seeding the viral supernatants treated with S-ONs collected after 72 h on 6-well plates containing Vero E6 cells and analyzing plaque reduction (PRA) at 3 days compared to the viral control grown in presence of lipofectamine in absence of S-ONs.

### 2.4. Cell Cytotoxicity Assay

The cytotoxicity of candidate S-ONs were evaluated by the MTT reduction assay. Vero E6 cells were plated at a density of 10^4^ cells per well in a flat-bottom 96-well culture plate and allowed to adhere overnight. When the cell layers were confluent, the medium was removed, the wells were washed twice with PBS and treated with 100 μL of MEM alone or with lipofectamine with or without the appropriate concentrations of the S-ONs under study (the final concentration added to the cells ranged from 0.1 to 100 μM) and incubated at 37 °C in a CO_2_ incubator for 72 h. After treatment, an MTT kit (Roche, Milan, Italy) was used according to the supplier’s instructions, and the absorbance of each well was determined using a microplate spectrophotometer at a wavelength of 595 nm. Cytotoxicity was calculated by dividing the average optical density of treated samples by the average of the mock-treated samples in the presence of lipofectamine alone.

### 2.5. RNA Extraction, Reverse Transcription, and Real-Time PCR of the Viral Genomic Positive and Negative Strand

RNA was extracted and purified from supernatant and infected cells using an RNAeasy mini Kit (Qiagen, Milan, Italy). One hundred nanograms of total RNA of each sample in a 20 μL reaction mixture were reverse-transcribed using the indicated primers (primer SC2-Rev 5′ CCT TGT GTG GTC TGC ATG AGT TTA G 3′ and RdRP_SARSr-R1 5′CAR ATG TTA AAS ACA CTA TTA GCA TA 3′, for the transcription of N and RdRp positive viral RNA respectively; primer SC2-For 5′ CCT TGT GTG GTC TGC ATG AGT TTA 3′, and RdRP_SARSr-F2 5′GTG ARA TGG TCA TGT GTG GCG G 3′ for the transcription of N and RdRp negative complementary viral RNA strand respectively; and primer 5′ TTT TTT TTT TTT 3′ for the transcription of viral and β-actin mRNA and amplified by real-time PCR using primers targeting the region of the N and RdRp viral and β-actin target (primer forward SC2-For 5′ CTG CAG ATT TGG ATG ATT TCT CC 3′, and reverse SC2-Rev 5′ CCT TGT GTG GTC TGC ATG AGT TTA G 3′ and probe SC2-Probe FAM-5′ ATT GCA ACA ATC CAT GAG CAG TGC TGA 3′-MGB for N; primer forward RdRP_SARSr-F2 5′GTG ARA TGG TCA TGT GTG GCG G 3′ and reverse RdRP_SARSr-R1 5′CAR ATG TTA AAS ACA CTA TTA GCA TA 3′ and probe RdRP_SARSr-P1 Fam-5′CCAGGTGGWACRTCATCMGGTGATGC 3′-MGB for RdRP; primer forward FPMGB 5′CCC GAT GGC CAG GTC A 3′, and reverse RP108 5′GGT AGT TTC ATG GAT GCC ACA G 3′ and probe MGBOV FAM-5′ CCA TTG GCA ATG AGC GG 3′-MGB for β-actin). Five microliters of each reverse-transcribed sample in 25 μl of the reaction volume was amplified in a Rotor-Gene Q real-time apparatus (Corbett research, Mortlake, Australia) using the PCR Master Mix (Life technologies, Foster City, CA, USA). The reaction was carried out at 95 °C for 10 min followed by 40 cycles (20 s at 95 °C, 60 s at 60 °C; the florescence was recorded at 75 °C). All reactions were performed in duplicate. Cycle times were calculated using the Rotor-Gene Q software version 2.3.1 and samples differing by 1.0 Ct unit between duplicates were discarded.

### 2.6. Immunofluorescence Investigation

Immunofluorescence microscopy was performed on methanol-acetone (1:1) fixed Vero E6 SARS-COV-2 infected cells treated with S-ON Cy5-labelled deoxyoligonucleotide. Cell labelling was carried out using an anti-M rabbit polyclonal antibody (MyBiosource, San Diego, CA, USA), an anti-N rabbit monoclonal antibody and anti-S mouse/human chimeric antibody (Sino Biological, Eschborn, Germany) followed by a secondary antibody conjugated to Alexa Fluor-488 (Jackson ImmunoResearch, Cambridge, UK). Nuclei were stained with 4,6-diamidino-2-phenylindole (DAPI). The images of cells were acquired by the inverted multi-channel fluorescence and transmitted light imaging EVOS™ M7000 Imaging System microscope (Invitrogen, Carlsbad, CA, USA) at a 40× magnification.

## 3. Results

### 3.1. Antiviral Activity of S-ON from the SL1 and SL2 Sequence in the 3′ End of the ORF1b against SARS-CoV-2

The S-ONs used in this study (Figure 1, panel A,B) were designed on the SARS-CoV-2 RNA stem-loop sequence SL1 and SL2 within the 3′ end of the ORF1b (panel A) previously identified as packaging sequences exhibiting homologies among SARS-CoV-2 variants and other CoVs (panel C) [23].The antiviral activity of the first 12 S-ON (13-19 mer) reproducing the direct and the complementary (antisense, As) genomic sequence, partially overlapped the SL1 and SL2 sequence (panel B), and of an S-ON scrambled form (SL Contr) are reported in Table 1.

Among the 12 S-ONs tested, several oligonucleotides exhibited a powerful dose dependent antiviral activity, showing fifty percent inhibitory concentration (IC_50_) ranging from 0.27 to 12 μM. It is of note that three S-ONs with high antiviral activity (SL1-2, SL1-2As and SL2-2 with IC_50_ of 0.27, 1.04 and 1.60 μM, respectively) overlapped the tip of the SL1 and SL2 and showed a secondary structure reproducing that of the target RNA genome. The S-ON with a scrambled sequence used in the same condition was not active. Importantly, under the experimental conditions used, neither did the S-ONs complex with lipofectamine at any concentration nor did lipofectamine alone exert a significant reduction in cell viability (Table 1), thus excluding the possibility that inhibition was merely a consequence of compound cytotoxicity.

To dissect the minimal sequence involved in the antiviral activity reported in Table 1, additional 12 S-ONs were synthetized with a reduced length (6-10 mer) and mutated in the central nt residues of the more active S-ONs (SL1-2, SL1-2 As, SL2-2, and SL2-2 As). Table 2 shows the antiviral activity of the short 12 S-ONs with an IC_50_ ranging from 0.19 to 31.12 μM. It is of note that the S-ONs with a length of 10-mer (SL1-4 and SL1-4AS, and SL2-4 and SL2-4AS) retained antiviral activity whereas when reduced to 6-mer (SL1-5, SL1-6, SL1-7, and SL2-5) a reduction in their effectiveness was observed. However, when the central region of the S-ON of 10-mer was completely mutated, its antiviral activity was lost (S-ON SL1-4mut and SL1-4ASmut, SL2-4mut, and SL2-4ASmut mutated form). Again, under the experimental conditions used, neither the S-ONs associated with lipofectamine at any concentration nor lipofectamine alone exerted cytotoxicity (Table 2). The results confirm that at least 10 nt of the loop region of SL1 and SL2 domain are essential for the antiviral activity.

The selected short 10-mer S-ONs (SL1-4, SL1-4As, SL2-4, SL2-4As) were assayed against different virus infectious doses. The selected S-ONs were effective also with different grades (MOI 0.001, 0.01 and 0.1) of virus infectious doses (IC_50_ ranged from 0.30 to 1.45 μM; see supplementary Figure S1) and exhibited a cell-independent antiviral activity (IC_50_ ranged from 0.51 to 1.64 μM and from 0.89 to 1.97 μM for Vero E6 and MCF-7-hACE2cells, respectively; see supplementary Figure S2)

Finally, the antiviral activity of the selected short 10-mer S-ONs (SL1-4, SL1-4As, SL2-4, SL2-4As) was assayed using different SARS-CoV-2 variants. To do this, although the SARS-CoV-2 RNA stem-loop sequence SL1 and SL2 within the 3′ end of the ORF1b showed the absence of nucleotide differences, it was considered of interest to investigate S-ON activity against selected variants of concern isolated during the pandemic. Table 3 reports that the S-ONs were active also with different, representative SARS-CoV-2 variants (IC_50_ range from 0.60 to 4.12 μM, from 1.23 to 2.34 μM and from 1.8 to 5.61 μM for Wuhan type variant, Alpha type variant and Delta type variant, respectively). It is of note that when the S-ONs were used against unrelated viruses, such as the influenza-virus-type A/H1N1 variant, the antiviral activity was totally absent.

### 3.2. Localization of S-ONs within Infected Cells

**Figure 2:**
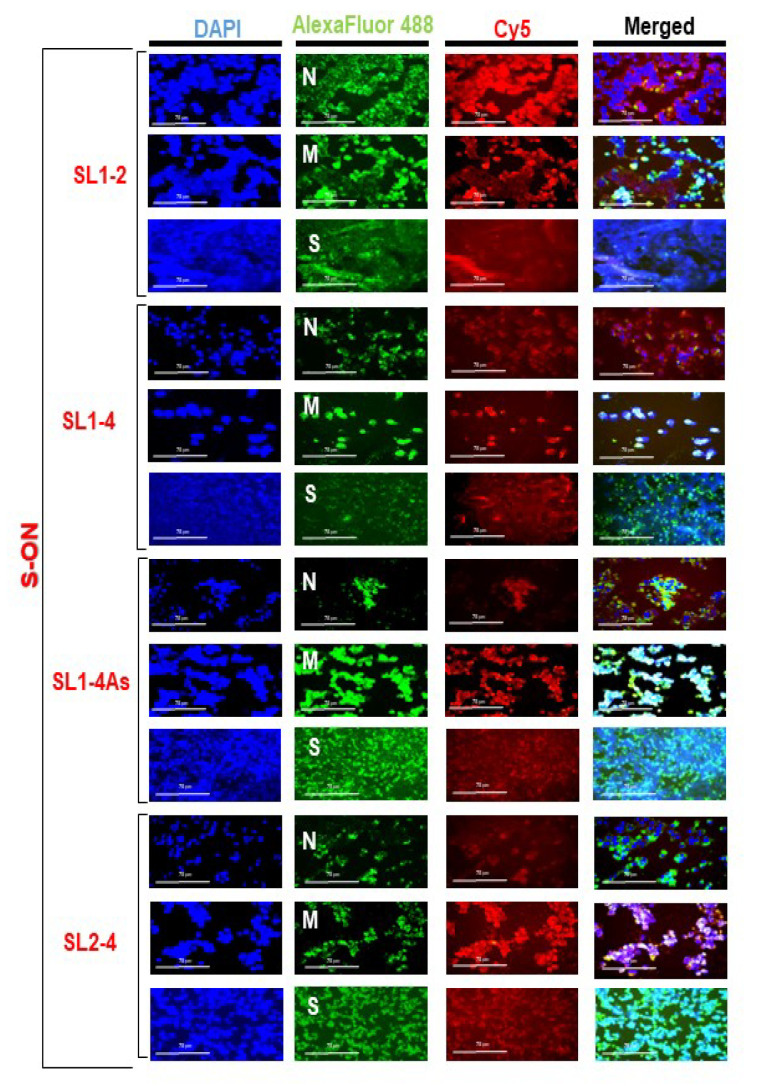
S-ONs and N, M, or S proteins’ intracellular localization in SARS-CoV-2 infected cells. Vero cells were infected with SARS-CoV-2 virus at a MOI of 0.01 and, after 1 h incubation, treated with the S-ONs under study at 10 μM (selected final concentration) using the standard protocol. Viral N, M, and spike protein expression was evaluated in infected cells after 24 h of infection. Immunofluorescence of Vero SARS-CoV-2 infected cells was performed with S-ON Cy5-labelled oligonucleotides and using primary antibodies (anti-M, anti-N, and anti-S SARS-COV-2 proteins followed with secondary antibodies conjugated to Alexa Fluor-488. After washing, nuclei were stained with 4,6-diamidino-2-phenylindole (DAPI). The images of cells were acquired by the inverted multi-channel fluorescence and transmitted light imaging with EVOS™ M7000 Imaging System microscope at a 40× magnification. The scale bars represent 75 μm.

Experiments to evaluate the intracellular localization of the S-ONs were performed. Vero E6 cells treated with S-ON Cy5-labelled deoxyoligonucleotide at different concentrations (0.1–10 μM, final concentration) were examined by immunofluorescence microscopy using the standard procedure. Figure S3 shows that the S-ONs exhibited a dose-dependent fluorescent signal inside the cells. Furthermore, selected S-ONs (SL1-2, SL 1-4, SL 1-4As and SL2-4) reported a predominant perinuclear/cytoplasmic localized fluorescent signal similar to that observed for M, N, and S SARS-CoV-2 proteins, within the cells (Figure 2).

### 3.3. Effect of Varying the Time of S-ON Treatment on Virus Inhibition

**Figure 3:**
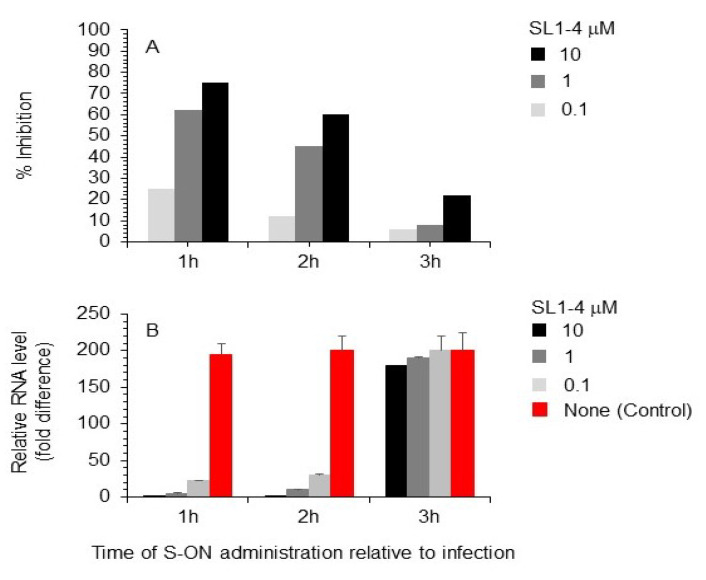
Effect of varying S-ON SL1-4 time of administration on SARS-CoV-2 replication. SARS-CoV-2 infection of Vero cells at MOI of 0.01 in presence of S-ON SL1-4 at 0.1, 1, and 10 μM selected concentration added at the indicated time (h) relative to the infection. (**A**). SARS-CoV-2 infection exposed to the indicated doses of S-ONs was assayed with the viral plaque-reduction assay. The values shown are means of 3 independent experiments. (**B**) The level of viral positive RNA extracted and purified from the supernatant of the same samples of the experiment in point (**A**) was measured by RT using N RNA-specific primers followed by real-time PCR as described in Materials and Methods (**B**). Values shown are means ± standard deviations of the relative levels of RNA obtained in 3 independent experiments.

To better evaluate the antiviral activity of our S-ONs, we selected S-ON SL1-4 as a representative of the series, to investigate the mechanism of action (Figure 3). This experiment was performed as described above except that cell cultures were treated with S-ON SL1-4 at selected times post-infection. The experiments with varied time of the S-ONs addition, performed at different SL1-4 concentrations, clearly demonstrated that S-ON acted in the first 2 h of the SARS-CoV-2 life cycle, since the addition of SL1-4 after 3 h post infection did not affect viral replication (Figure 3).

### 3.4. Effect of S-ON Treatment on Viral RNA Replication and M, N Protein Expression at Different Times of Infection

**Figure 4:**
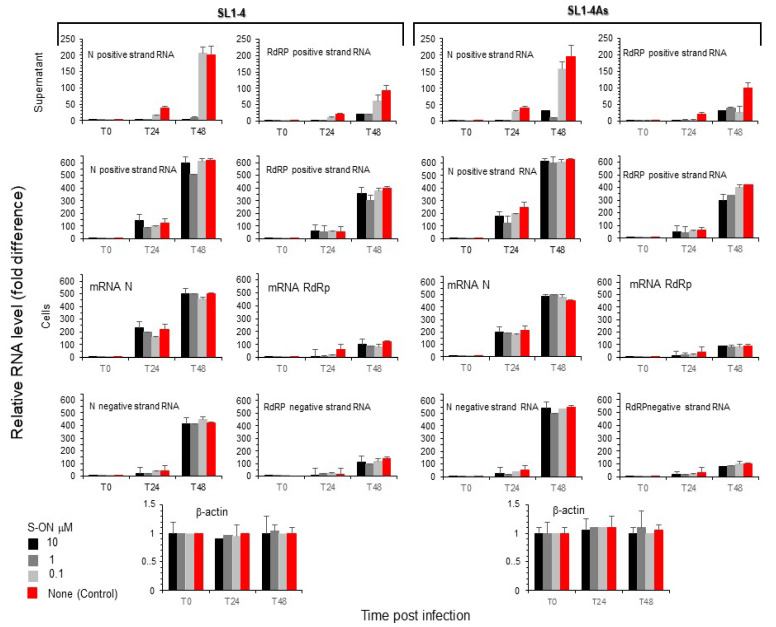
Effect of S-ON treatment on viral RNA replication. Vero cells were infected with SARS-CoV-2 virus at a MOI of 0.01 and, after 1 h incubation, treated with the S-ONs under study at 10 μM (selected final concentration) using the standard protocol. Viral RNA was extracted and purified from the supernatant and infected cells immediately (T0), and after 24 h (T24) and 48 h (T48) of infection. The levels of N- and RdRp-specific viral positive strand RNA, viral negative complementary RNA, and viral messenger RNA were measured by RT using RNA-specific primers followed by real-time PCR as described in Materials and Methods. The level of β-Actin mRNA in the same sample was also measured and used to normalize viral cells’ RNA levels. Values shown are means ± standard deviations of the relative levels of RNA obtained in 3 independent experiments.

**Figure 5:**
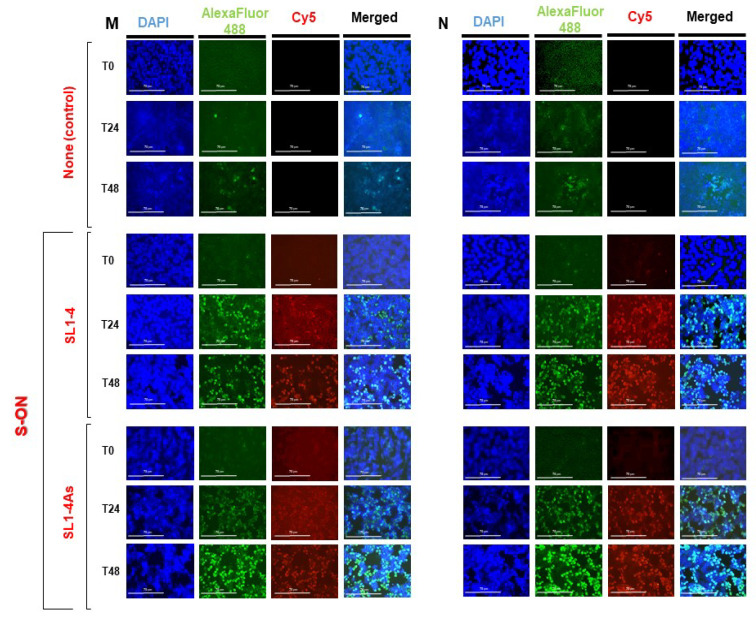
Effect of S-ONs treatment on viral N and M protein expression. Vero cells were infected with SARS-CoV-2 virus at an MOI of 0.01 and, after 1 h incubation, treated with the S-ONs under study at 10 μM (selected final concentration) using the standard protocol. Viral N and M protein expression was evaluated in infected cells immediately (T0), and after 24 h (T24), 48 h (T48) of infection. Immunofluorescence of Vero SARS-CoV-2 infected cells was performed with S-ON Cy5-labelled compound and using primary antibodies (anti-M and anti-N SARS-CoV-2 proteins) followed with secondary antibodies conjugated to Alexa Fluor-488. After washing, nuclei were stained with 4,6-diamidino-2-phenylindole (DAPI). The images of cells were acquired by the inverted multi-channel fluorescence and transmitted light imaging using an EVOS™ M7000 Imaging System microscope at a 40× magnification. The scale bars represent 75 μM.

We looked at the understanding that the observed inhibition of virus production was related to the interference of S-ON with the replication of the viral RNA. First, we examined a viral positive RNA strand in the supernatant immediately (T0) and after 24 h (T24) and 48 h (T48) of infection using a specific reverse-transcription and real-time PCR assay. As reported in Figure 4, a significant reduction in relative RNA levels during S-ONs treatment was observed for both SL1-4 and SL1-4As molecules after 24 h of infection. We examined the accumulation of the positive and negative strand RNA species produced in Vero cells treated with SL1-4 and SL1-4As. In this context, a significant difference for both types of viral RNA species was not observed with the treatment of SL1-4 and SL1-4As (Figure 4). Subsequently, it was considered of interest to examine, at the same time post infection, the presence of N and M protein expression within the infected cells. Figure 5 (see also supplementary Figures S4 and S5) shows that, compared to the infected cells treated with virus and lipofectamine alone, during treatment with both SL1-4 and SL1-4As S-ON, viral M and N protein expression was not significantly reduced. Conversely, a high accumulation of M and N protein at 48h post infection was observed in the cells treated with both S-ONs compared to those in virus control alone that was absent in the low S-ON concentrations (Figure 5 and supplementary Figure S4 and S5).

## 4. Discussion

The present study investigated whether the sequences within the SL1 and SL2 stem-loop packaging sequence in the 3′-end of the ORF1b SARS-CoV-2 genomic RNA are suitable targets for the development of novel antiviral compounds. In this effort, dissecting the minimal sequence involved in the antiviral activity, 10-mer S-ONs with a conserved powerful antiviral activity (SL1-4 and SL1-4AS, and SL2-4 and SL2-4AS) overlapping the tip of the SL1 and SL2 were identified. The effect was sequence-specific, since a scrambled version of S-ON was completely devoid of activity. Moreover, when the central region of the S-ON of 10-mer was completely mutated; its antiviral activity was lost. Under the experimental conditions used, neither the S-ONs complexed with lipofectamine at any concentration nor lipofectamine alone exerted cytotoxicity. The inhibition of SARS-CoV-2 virus replication by short S-ONs (SL1-4, SL1-4As, SL2-4, SL2-4As) was proved to be dose-dependent and independent of the multiplicity of infection. Furthermore, the antiviral activity of the selected short S-ONs was active also with different representative SARS-CoV-2 variants but ineffective when the S-ONs were used against an unrelated virus, such the influenza-virus-type A/H1N1 variant. Thus, for the conservation of the target sequence of S-ONs among SARS-CoV-2 variants, although not confirmed experimentally in this study, it is likely that S-ONs may be active also for all new recent SARS-CoV-2 variants (omicron-like).

To date, several efforts have been undertaken to develop synthetic oligonucleotides, using several modifications (such as phosphorotioate (S) backbone modification, 2′-methoxyethyl (2′- ribose substitution (MOE), locked nucleic acid (LNA) conformationally constrained analogues, and phosphorodiamidate morpholino (PMO) alternative chemistries), targeting viruses including SARS-CoV-2 as new antivirals [35,36,37,38]. FDA-approved antisense oligonucleotide therapies are made up with 18–30-mer compounds designed on cis-acting protein-expression gene features [38]. Despite a breadth of knowledge about the viral life cycle, the understanding of the RNA secondary structure of the genome is in its infancy. Research on other RNA viruses has revealed that genomic RNA is capable of playing many important roles in viral lifecycles beyond merely encoding amino acid sequences, suggesting that viral RNA structural elements could be promising therapeutic targets [6,9,11]. Among these, packaging signals used by viruses to deliver their own genome to viral particles have been extensively used [12]. In this context, for influenza virus studies a powerful S-ON antiviral activity has been proved [17,18,19]. Additionally, a small interfering RNA (siRNA) was developed for treating influenza virus infections in vivo and in vitro [20,39]. It is of note that experimental evidence reported that targeting packaging signals with nucleic acid-based antivirals may be difficult for the virus to evade through resistance mutations [20].

A peculiarity of the present study is the fact that a 10-mer S-ONs maintains antiviral activity confirming the uniqueness of SL1 and SL2 target studied. To support our data, recent studies, using quantitative RNA structure analysis at single nucleotide resolution, suggested that shorter oligonucleotides designed on the fundamental RNA structure implicated in viral life cycle may be able to achieve sufficient affinity and specificity interactions with targets [40,41].

At this time, it is difficult to speculate on the molecular mechanism(s) by which SL1-4 and SL2-4 S-ON block SARS-CoV-2 virus replication. The results obtained using microscopy coupled with the experiments in which the inhibitor was added at different time of virus infection suggested that intracellular uptake of S-ONs, to obtain an inhibitory effect, was already efficiently achieved after 1 h of treatment of the test cultures. It is of note that the localization of S-ON was predominantly cytoplasmic with partial co-association of N and M proteins. However, the antiviral activity of S-ONs proved to be time-dependent, as revealed by the experiments in which the inhibitor exerted a clear-cut reduction in virus replication only when added to the test cultures within 2 h of virus infection. Although showing different temporal accumulation during viral infection, the relative levels of viral RNA positive and negative strand species corresponding to the N and RdRP genes observed after 24 and 48 h of infection showed a significant difference in the supernatant but did not differ in infected cells treated with S-ONs compared to the control infected cells in the absence of these molecules. Additionally, the presence of N and M protein positive signal accumulation was high in the infected cells treated with S-ON and increased during infection. Thus, these results suggest that the activity of the S-ONs is not related to an impairment in the intracellular accumulation of viral RNA but to an inhibition in a different stage of viral replication, reducing viral progeny formation. The clear tendency of S-ON to localize in the cell cytoplasmically/perinuclearly suggests that its inhibitory activity may be directed to target(s) belonging to this cellular compartment, in which all viral RNA segments and proteins involved in the subsequent step of viral formation would be available for interaction with it. Usually, the S-ON antisense technology works by binding to a target-RNA-activating RNAase H with the degradation of the RNA-DNA heteroduplex, or by binding to a target RNA, impairing RNA processing and the interactions of the target RNA with key proteins [9]. The observation that S-ONs and their antisense counterpart exerted an inhibitory activity suggests that these molecules might act also by counteracting the interaction(s) of viral RNA with other viral RNA and/or proteins. Hence, a possible explanation might be that S-ON interacting with the RNA or protein (N and/or M) that contains the target site of endogenous viral RNA and S-ONsAs pairing directly with the packaging-signal region of the ORF1b genome RNA (with its consequent degradation or impaired function) hampers the molecular interaction needed for infectious virus production in both cases [42,43]. However, identifying the precise way by which the inhibition of virus replication is achieved will require further studies.

Progress in oligonucleotide chemistry to improve the drug properties and reduce cost has paved the way to the development of oligonucleotide antiviral molecules. The recent development of new technology, from computation analysis to RNA structural molecular investigation, paved the way for important discovery platforms for the development of innovative therapeutic nucleic acids in addition to small molecules and antibody therapeutics [44]. Moreover, the short nature of oligonucleotides designed in these structural investigations, allowing their more efficient delivery and favorable bio-distribution, and the adoption of delivery technologies, such as conjugates or nanoparticles, have been game changers for many therapeutic indications. Indeed, evidence suggests that new nanoparticle delivery systems, including the use of extracellular vesicles, have increased the efficiency of nucleic acid delivery into the host [38,44].

Collectively, targeting the SARS-CoV-2 packaging region in the 3′ end of ORF1b, which is highly conserved among SARS-CoV-2 viruses and plays a dominant role in the correct assembly of virions, makes it an extremely interesting target for the development of new therapeutics to deal with these ever-changing viruses [4]. Further investigation will be needed for the development of nucleic-acid viral interfering antiviral therapy.

## Figures and Tables

**Table 1:** Antiviral Activities and Cytotoxicity of S-ONs against SARS-CoV-2.

ID S-ON	Sequence (nt Length)	IC_50_ μM	CC_50_ μM	Secondary Structure
		Mean ± SD		
SL1-1	*attggtgtttgttctatga (19 nt)*	5.69 ± 1.20	>100	None
SL1-1As	*taaccacaaacaagatact (19 nt)*	12.21 ± 2.30	>100	None
SL1-2	*ttctatgactgacatagcc (19 nt)*	0.27 ± 0.18	>100	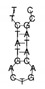
SL1-2As	*aagatactgactgtatcgg (19 nt)*	1.04 ± 0.81	>100	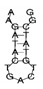
SL1-3	*acatagccaagaaaccaac (19 nt)*	5.80 ± 2.32	>100	None
SL1-3As	*tgtatcggttctttggttg (19 nt)*	2.90 ± 1.23	>100	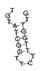
SL2-1	*actcactgtcttt (13 nt)*	7.23 ± 1.21	>100	None
SL2-1As	*tgagtgacagaaa (13 nt)*	9.90 ± 2.02	>100	None
SL2-2	*actgtcttttttgatggta (19 nt)*	1.60 + 0.89	>100	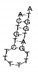
SL2-2As	*tgacagaaaaaactaccat (19 nt)*	2.60 ± 2.19	>100	None
SL2-3	*ttgatggtagagt (13 nt)*	4.48 ± 2.34	>100	None
SL2-3As	*aactaccatctca (13 nt)*	1.90 ± 1.20	>100	None
SL Contr	*atttcgatcaagacgctct (19 nt)*	>100	>100	None

IC_50_, fifty percent inhibitory concentration; CC_50_, fifty percent cytotoxicity concentration; SD, standard deviation. The oligonucleotide secondary structure was obtained using Mfold structure-prediction system [34].

**Table 2:** Antiviral activities and cytotoxicity of selected S-ONs against SARS-CoV-2.

ID S-ON	Sequence (nt Length)	IC_50_ μM	CC_50_ μM
		Mean ± SD	
SL1-4	*atgactgaca (10 nt)*	0.19 ± 0.20	>100
SL1-4As	*tactgactgt (10 nt)*	0.30 ± 0.29	>100
SL1-5	*gactga (6 nt)*	2.1 ± 1.23	>100
SL1-6	*actgac (6 nt)*	9.80 ± 10.11	>100
SL1-7	*ctgaca (6 nt)*	2.80 ± 1.70	>100
SL1-4mut	*atggggggca (10 nt)*	>100	>100
SL1-4Asmut	*tagggggggt (10 nt)*	>100	>100
SL2-4	*tcttttttga (10 nt)*	2.87 ± 2.30	>100
SL2-4As	*agaaaaaact (10 nt)*	0.93 ± 0.12	>100
SL2-5	*tttttt (6 nt)*	31.12 ± 9.05	>100
SL2-4mut	*tcggggggga (10 nt)*	>100	>100
SL2-4Asmut	*agggggggct (10 nt)*	>100	>100

IC_50_, fifty percent inhibitory concentration; CC_50_, fifty percent cytotoxicity concentration; SD, standard deviation.

**Table 3:** Antiviral activities of selected S-ONs against SARS-CoV-2 variants and unrelated virus.

ID S-ON	Sequence (nt Length)	IC_50_ μM	CC_50_ μM
		Mean ± SD	
Wild type-like			
SL1-4	*atgactgaca (10 nt)*	4.12 ± 2.23	>100
SL1-4As	*tactgactgt (10 nt)*	2.10 ± 2.09	>100
SL2-4	*tcttttttga (10 nt)*	1.50 ± 1.20	>100
SL2-4As	*agaaaaaact (10 nt)*	0.65 ± 0.45	>100
Alpha-like variant			
SL1-4	*atgactgaca (10 nt)*	1.23 ± 0.87	>100
SL1-4As	*tactgactgt (10 nt)*	2.10 ± 1.09	>100
SL2-4	*tcttttttga (10 nt)*	2.34 ± 1.11	>100
SL2-4As	*agaaaaaact (10 nt)*	1.90 ± 0.88	>100
Delta-like variant			
SL1-4	*atgactgaca (10 nt)*	5.61 ± 2.34	>100
SL1-4As	*tactgactgt (10 nt)*	4.64 ± 1.24	>100
SL2-4	*tcttttttga (10 nt)*	5.43 ± 3.32	>100
SL2-4As	*agaaaaaact (10 nt)*	1.88 ± 0.98	>100
A/Firenze/02/2019 H1N1pmd			
SL1-4	*atgactgaca (10 nt)*	>100	>100
SL1-4As	*tactgactgt (10 nt)*	>100	>100
SL2-4	*tcttttttga (10 nt)*	>100	>100
SL2-4As	*agaaaaaact (10 nt)*	>100	>100

IC_50_, fifty percent inhibitory concentration; CC_50_, fifty percent cytotoxicity concentration; SD, Standard deviation.

## Data Availability

Not applicable.

## Supplementary Materials

The following supporting information can be downloaded at: https://www.mdpi.com/article/10.3390/pathogens11111286/s1.
