# Supplementary figures and images for: Antiviral Activity of Oligonucleotides Targeting the SARS-CoV-2 Genomic RNA Stem-Loop Sequences within the 3′-End of the ORF1b
Source: Pathogens. 2022 Nov 1;11(11):1286. doi: 10.3390/pathogens11111286 (PMC9696570; doi:10.3390/pathogens11111286)

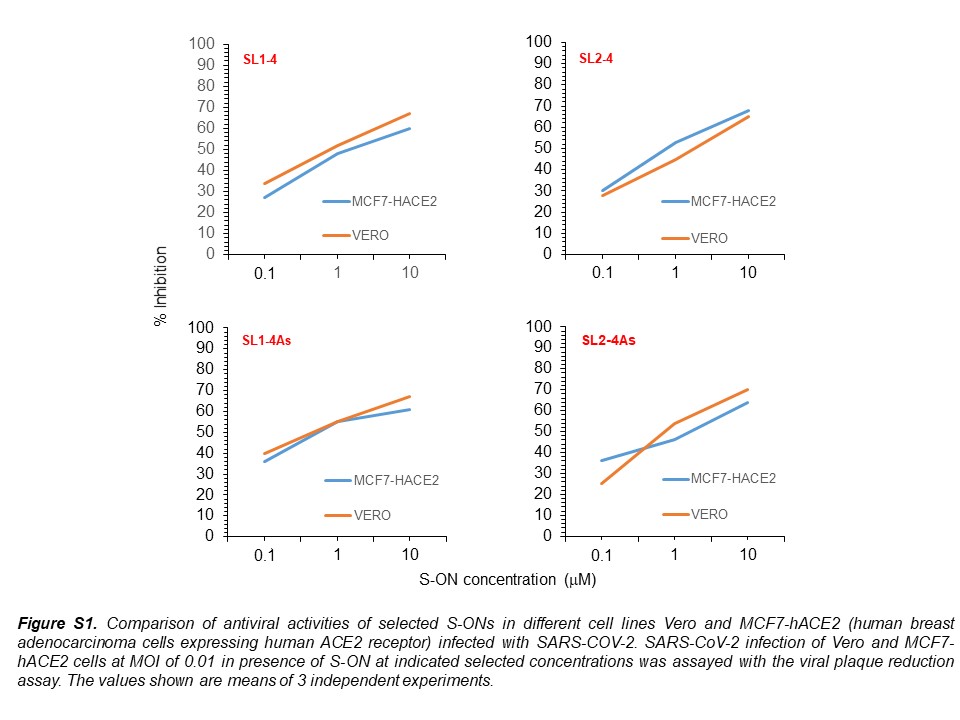

Supplement: Supplementary file 1 [file pathogens-11-01286-s001.zip › Figures S1-S5/Figure S1.JPG]

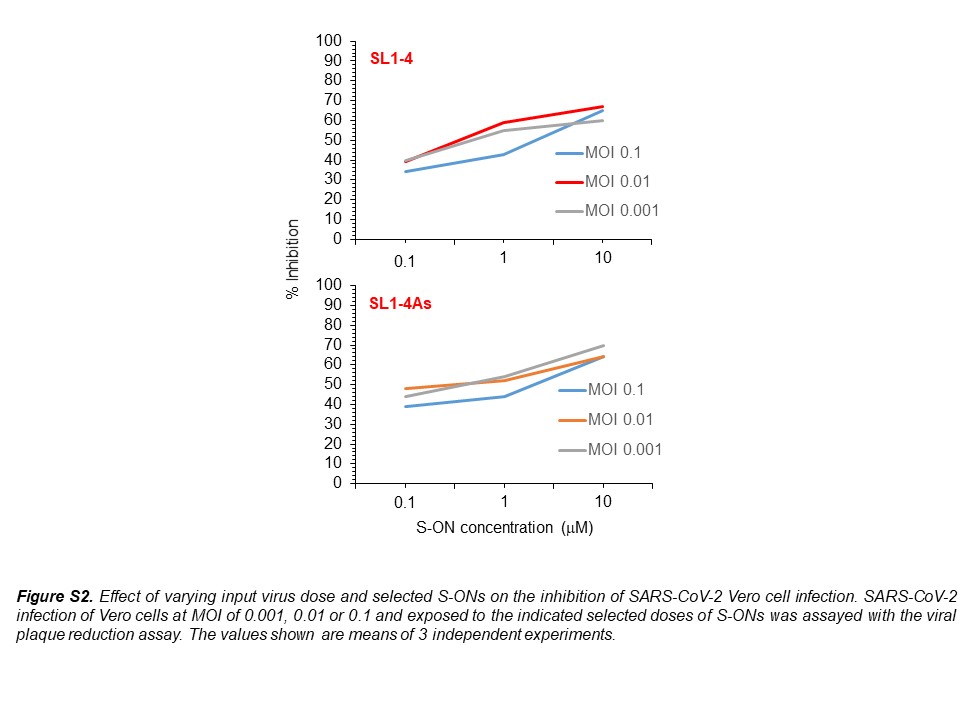

Supplement: Supplementary file 1 [file pathogens-11-01286-s001.zip › Figures S1-S5/Figure S2.JPG]

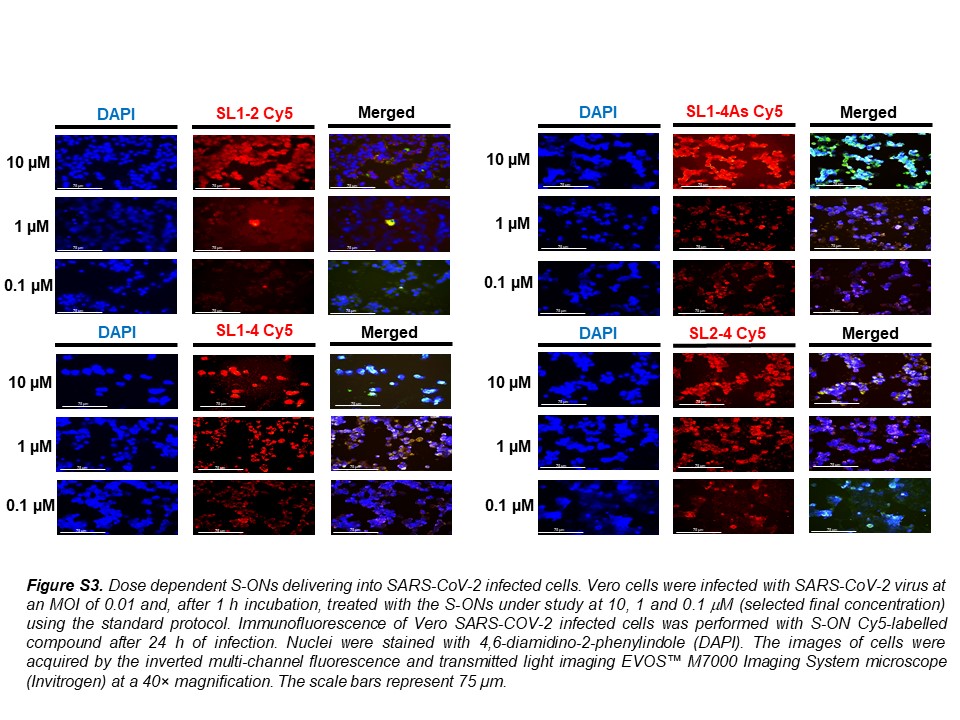

Supplement: Supplementary file 1 [file pathogens-11-01286-s001.zip › Figures S1-S5/Figure S3.JPG]

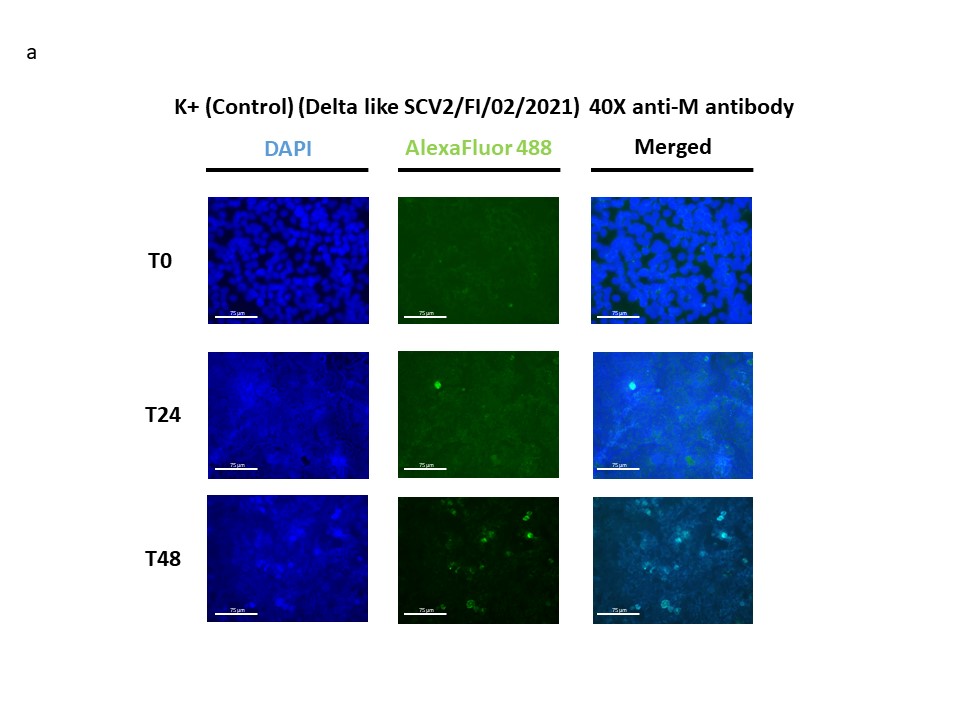

Supplement: Supplementary file 1 [file pathogens-11-01286-s001.zip › Figures S1-S5/Figure S4a.JPG]

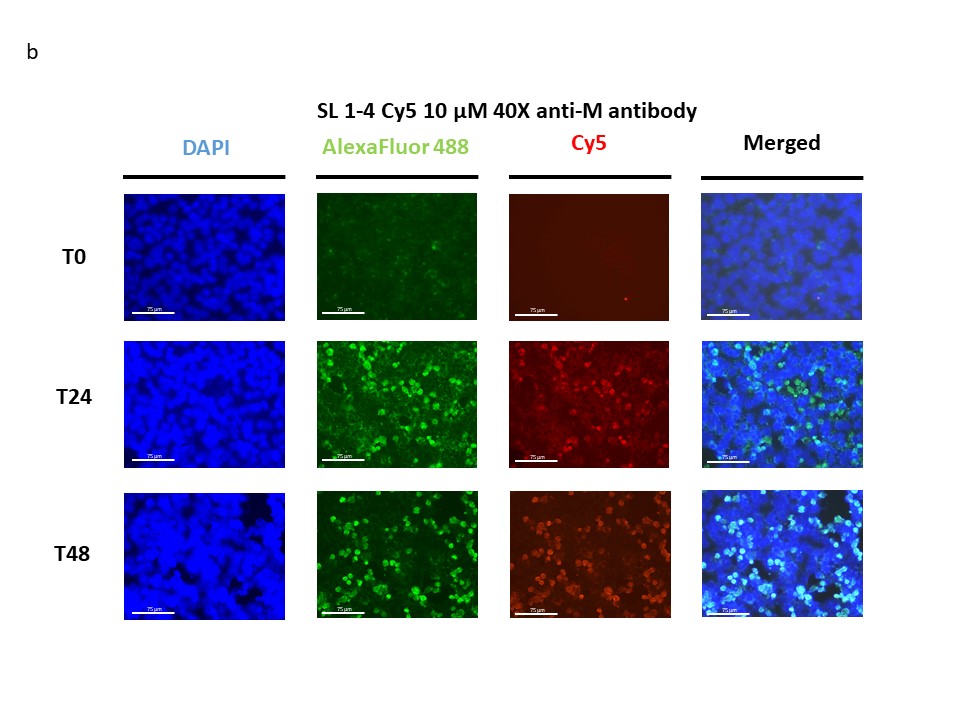

Supplement: Supplementary file 1 [file pathogens-11-01286-s001.zip › Figures S1-S5/Figure S4b.JPG]

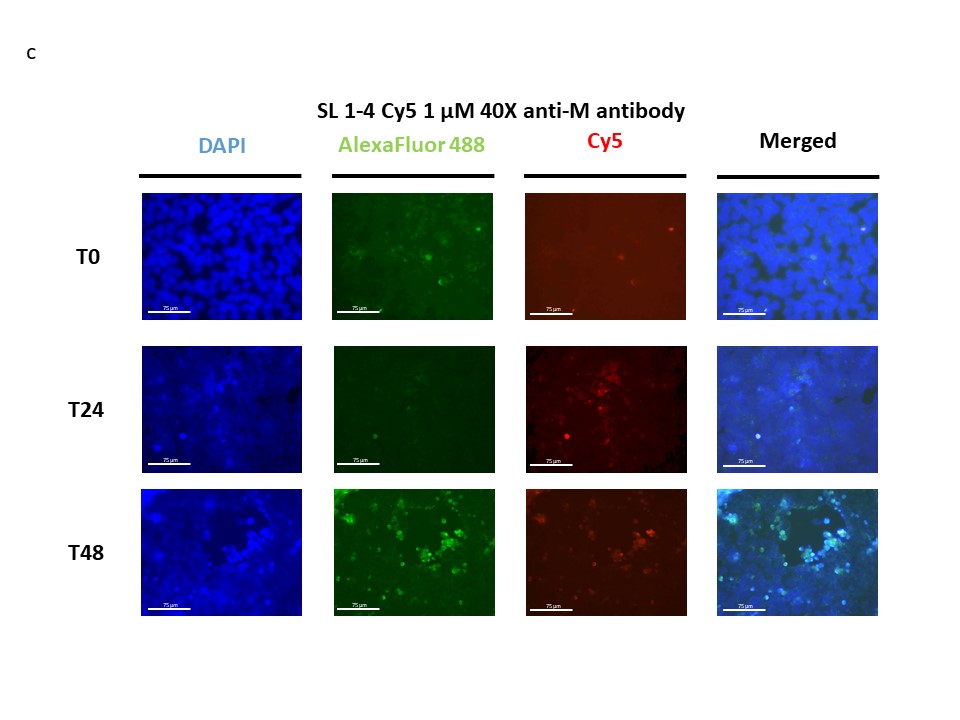

Supplement: Supplementary file 1 [file pathogens-11-01286-s001.zip › Figures S1-S5/Figure S4c.JPG]

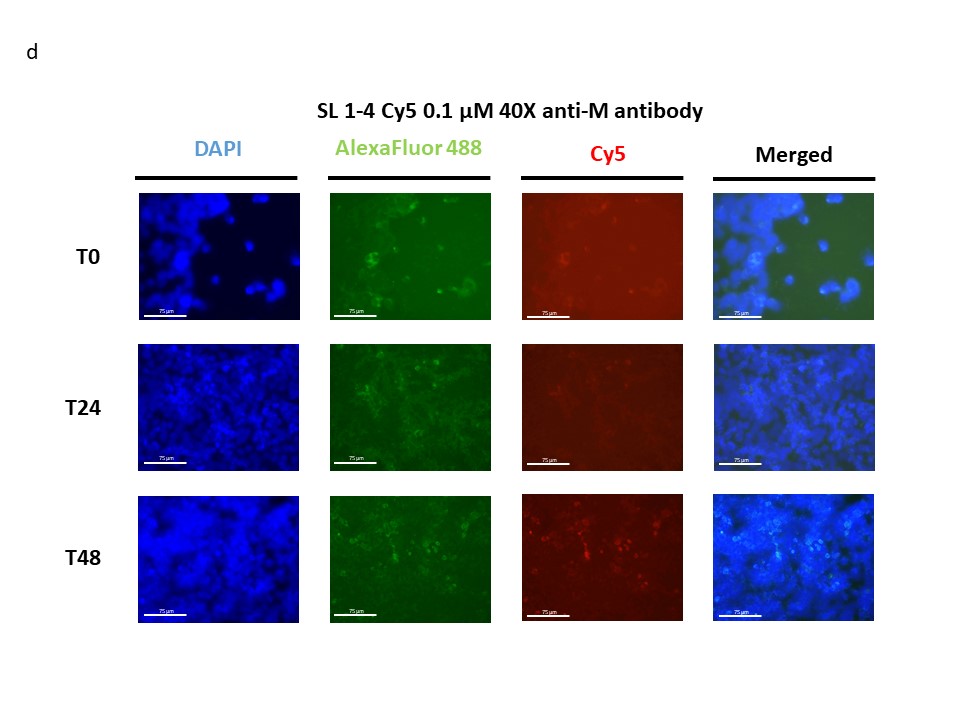

Supplement: Supplementary file 1 [file pathogens-11-01286-s001.zip › Figures S1-S5/Figure S4d.JPG]

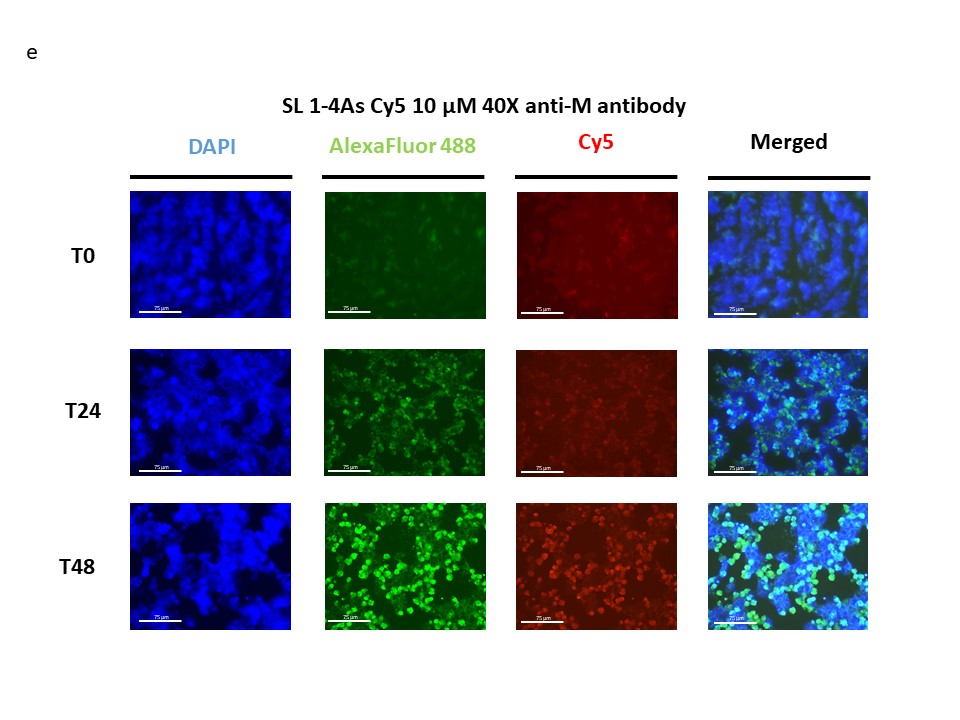

Supplement: Supplementary file 1 [file pathogens-11-01286-s001.zip › Figures S1-S5/Figure S4e.JPG]

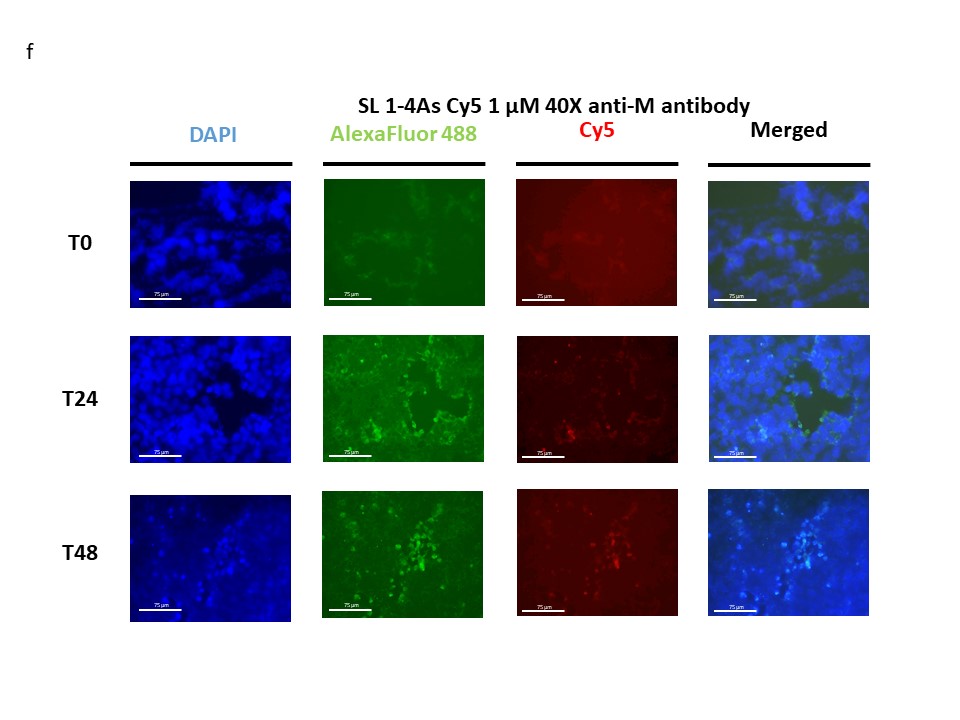

Supplement: Supplementary file 1 [file pathogens-11-01286-s001.zip › Figures S1-S5/Figure S4f.JPG]

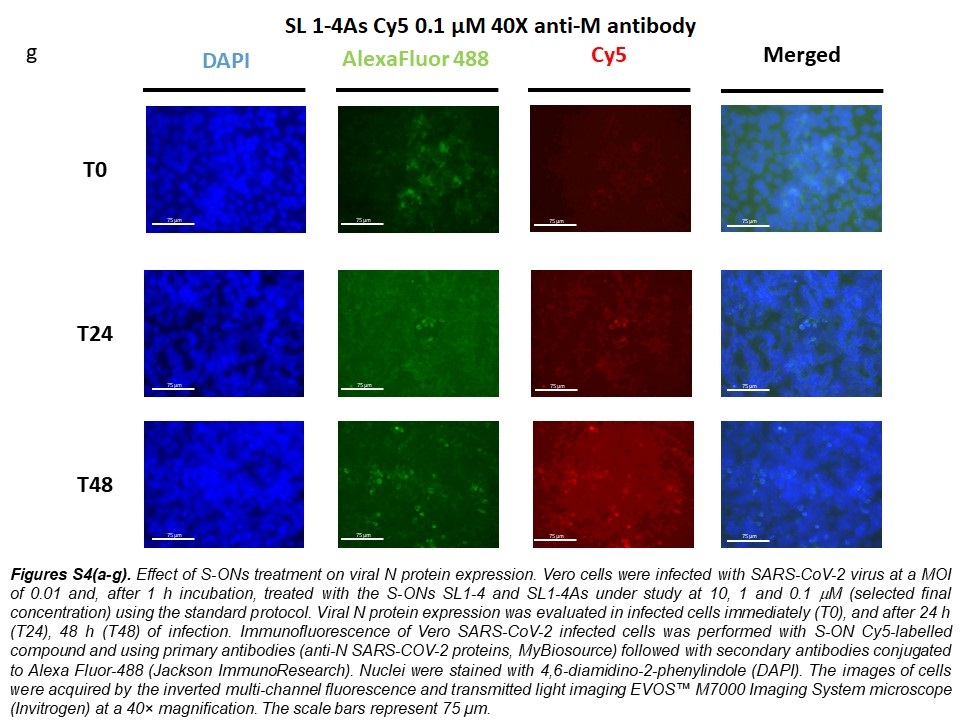

Supplement: Supplementary file 1 [file pathogens-11-01286-s001.zip › Figures S1-S5/Figure S4g.JPG]

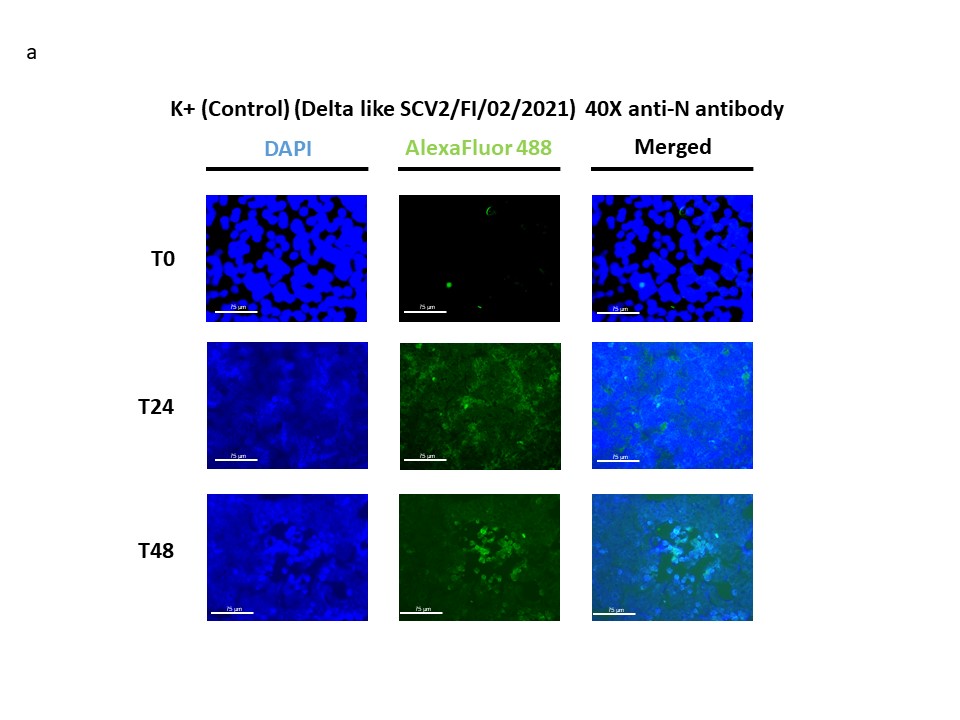

Supplement: Supplementary file 1 [file pathogens-11-01286-s001.zip › Figures S1-S5/Figure S5a.JPG]

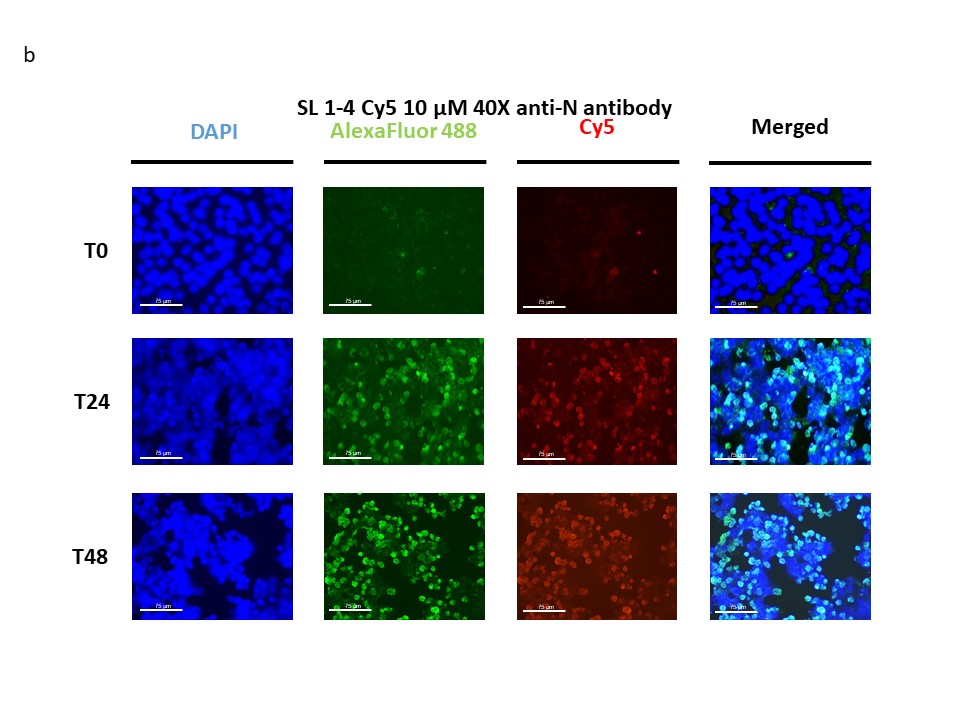

Supplement: Supplementary file 1 [file pathogens-11-01286-s001.zip › Figures S1-S5/Figure S5b.JPG]

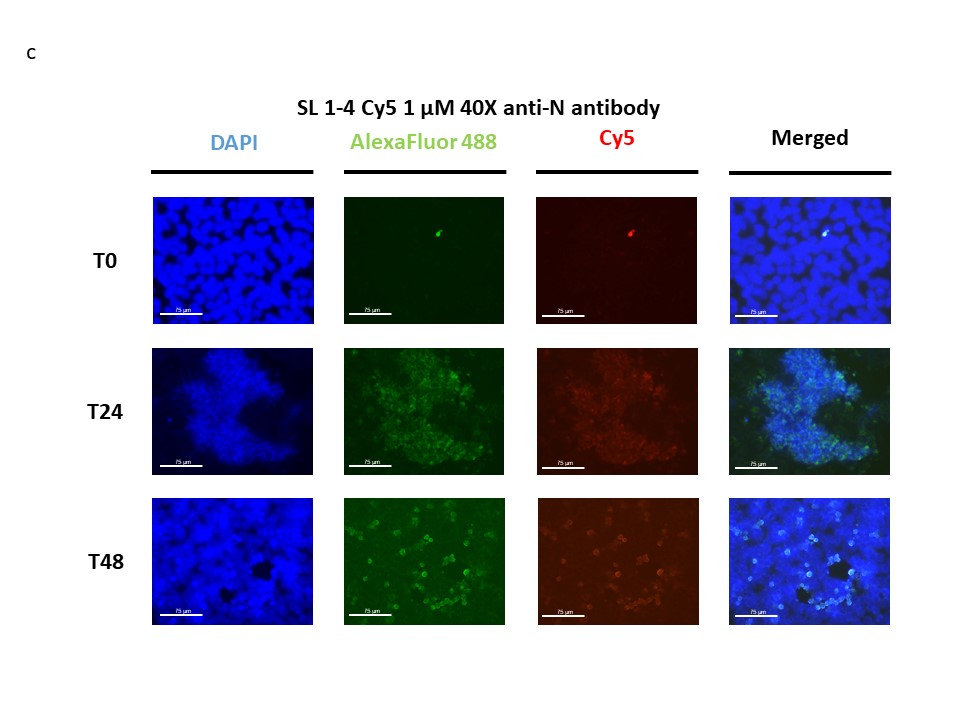

Supplement: Supplementary file 1 [file pathogens-11-01286-s001.zip › Figures S1-S5/Figure S5c.JPG]

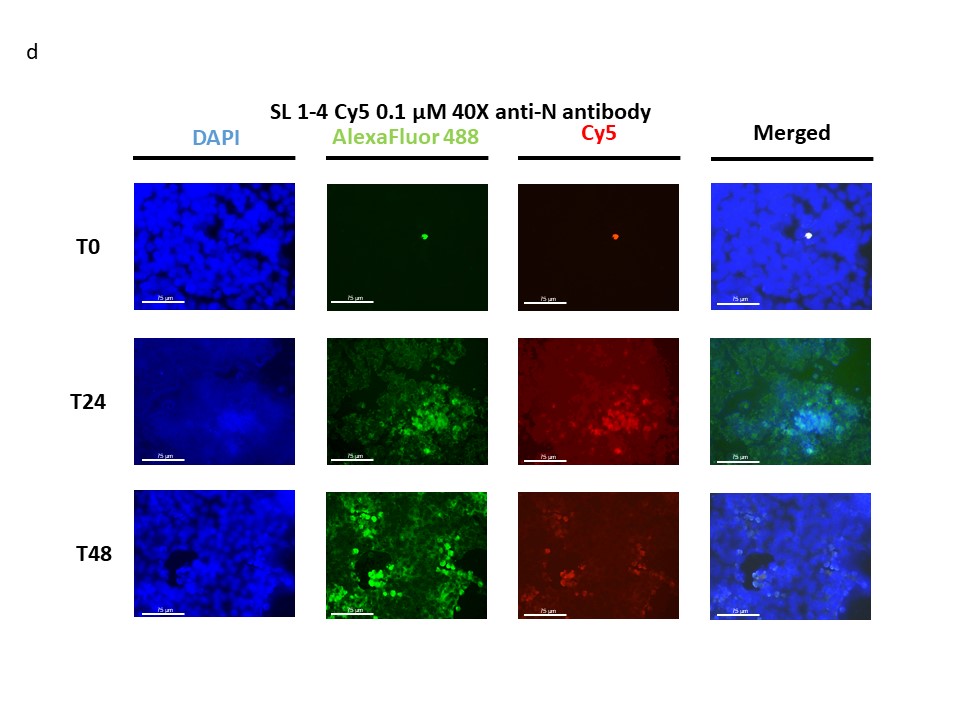

Supplement: Supplementary file 1 [file pathogens-11-01286-s001.zip › Figures S1-S5/Figure S5d.JPG]

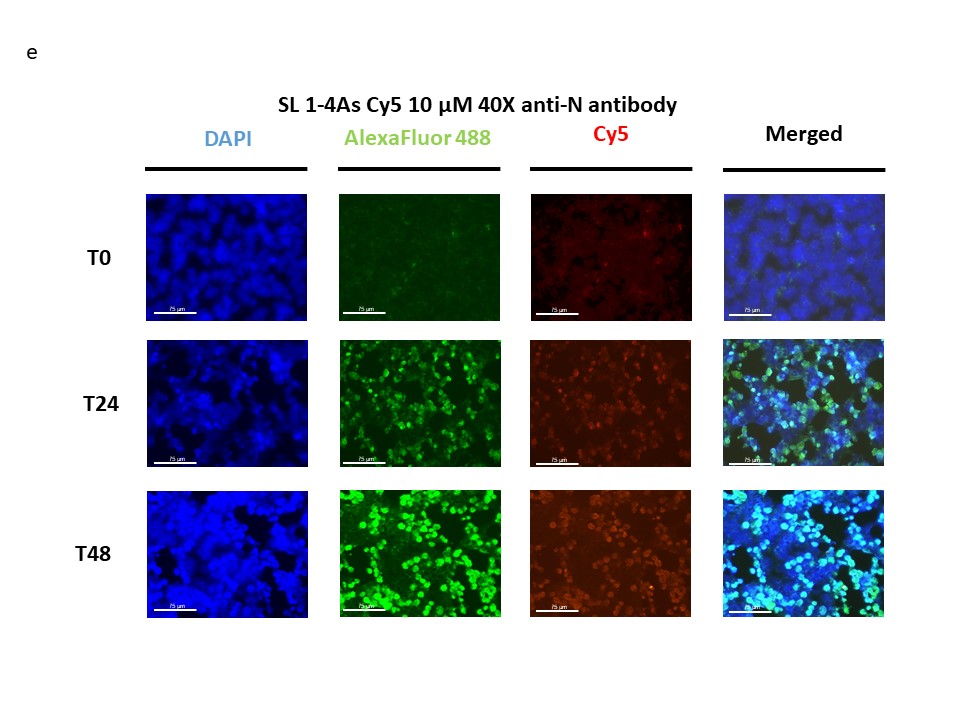

Supplement: Supplementary file 1 [file pathogens-11-01286-s001.zip › Figures S1-S5/Figure S5e.JPG]

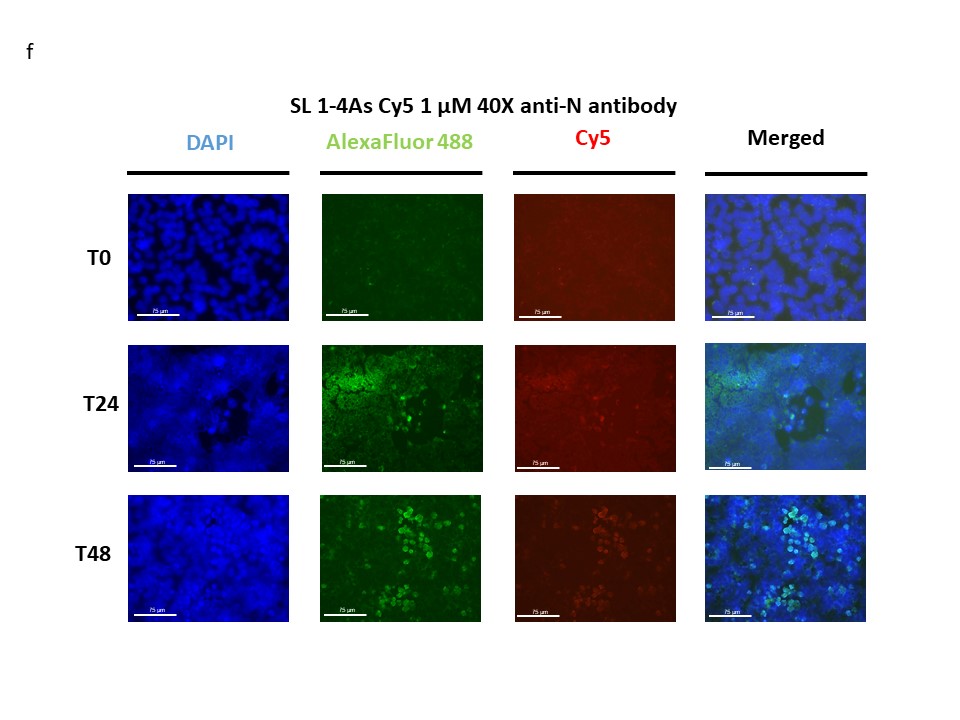

Supplement: Supplementary file 1 [file pathogens-11-01286-s001.zip › Figures S1-S5/Figure S5f.JPG]

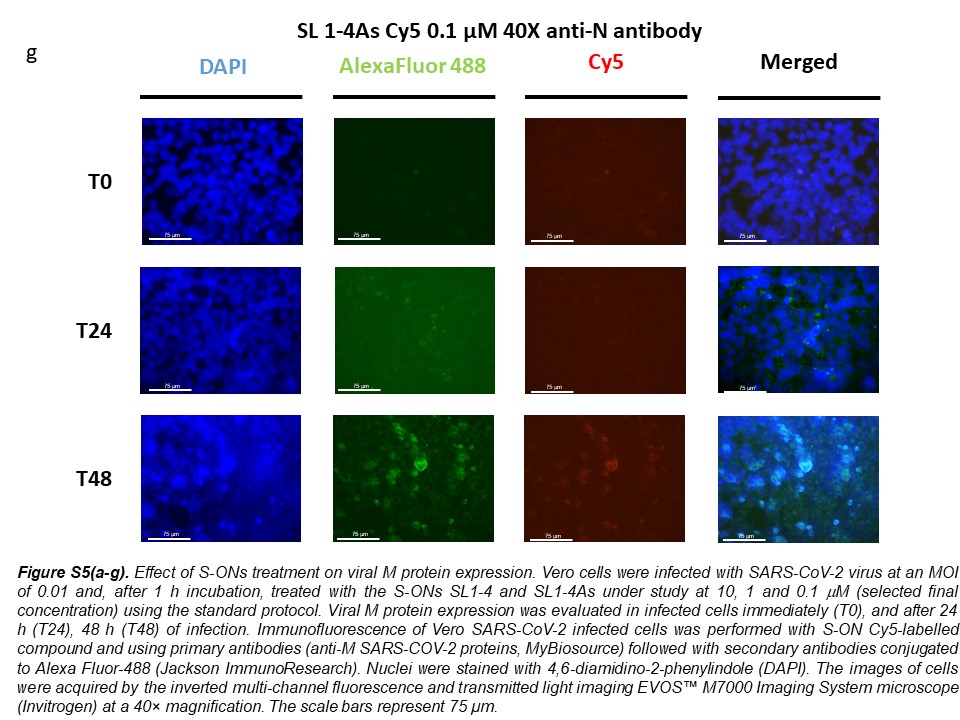

Supplement: Supplementary file 1 [file pathogens-11-01286-s001.zip › Figures S1-S5/Figure S5g.JPG]
